# 

**Published:** 1891-07

**Authors:** 


### MEETINGS.

GRAND RAPIDS, June 22, 1891.

DEAR DOCTOR: The thirty-sixth annual meeting of the Michigan Dental Association, will be held at Sault Ste. Marie, August 18, 19 and 20th, 1891. Special rates by rail and boat are being arranged, and the prospects are for a very large attendance. We are desirous of preparing a full programe for the meeting, which will be mailed to the members in a few weeks, and to this end request an immediate response from all members who propose to contribute to the interest of the meeting either by a paper or a clinic. Yours truly,

J. WARDHOUSE, Secretary.

Room 2. Widdicomb Building.

---

THE American Dental Society of Europe will hold its seventeenth annual meeting at Heidelberg on the Neckar, in the beautifully situated Schloss Hotel, on August third, fourth and fifth, 1891.

The officers for the year are:

*President:* Dr. William R. Patton, Cologne.
*Vice-President:* Dr. Isaac B. Davenport, Paris.
*Treasurer:* Dr. Charles H. Adams, Frankfort.
*Secretary:* Dr. Lyman C. Bryan, Basel.

EXECUTIVE COMMITTEE:

Drs. Patton, Adams and Wetzel.

MEMBERSHIP COMMITTEE:

Drs. Davenport, Jenkins and Prof. Miller.

Members of the profession are cordially invited, and are requested to notify the Secretary at an early date of their intention to attend the meeting, contribute papers or demonstrate before the Society. Programmes will be issued by June first and may be had on application. The charming site of Heidelberg will allow the Society to intersperse its three days proceedings with excursions to interesting points and visiting the University and the magnificent ruins of the castle.

### Meeting of the Committees on Dental Patents.

There will be a meeting held at the Town Hall, Saratoga, Monday evening, Aug. 3d, at eight o'clock, of all the committees appointed by the different dental societies all over the United States for the purpose of organizing and taking some action toward the prevention of further issuing of patents upon operations in the mouth. It is hoped that every society will see to it that they are represented at this meeting. Remember it will be held the Monday evening preceding the meeting of the American Dental Association. S. C. G. WATKINS, Chairman for New Jersey Committee, W. L. FISH, Secretary.

### EDITORIAL.

### The American Dental Association.

### CONTENTS.

COMMUNICATIONS.

A MONTHLY MAGAZINE DEVOTED TO THE INTERESTS OF THE

### DENTAL PROFESSION.

*Terms Reduced to* $2.00 *Per Year.* *Single Copies,* 25 *Cents.*

### EDITED BY PROF. J. TAFT, D.D.S.

—AND—

### Published by SAM'L A. CROCKER & CO., Ohio Dental and Surgical Depot.

The volume commences in January and closes in December.

The REGISTER being issued monthly is always fresh, therefore Indispensable to the practitioner who desires to keep posted up with the new inventions and improvements which are daily developed. Neither expense nor effort will be spared to give value and variety to its contents. Articles will be illustrated with well executed engravings whenever they will add to the interest or assist to explanation.

Its extensive circulation and frequency of issue make it one of the BEST DENTAL ADVERTISING MEDIUMS in the United States.

All subscriptions must begin with January or July.

Local or special notices, five lines or less, will be inserted for $3 each insertion; over five lines, 50 cts. per line.

All advertising bills payable in advance, or at furthest, after the issue of the first number containing the advertisement. All transient advertisements to be paid for in advance.

☞CONTRIBUTIONS SOLICITED.

Exclusively Editorial Communications should be addressed to the Editor.

The latest number of the REGISTER may be ordered with or without other goods at any time, and all letters should be addressed to

SAM'L A. CROCKER & CO., Ohio Dental and Surgical Depot, Cincinnati, O.

